# Supplementary material for: Content-rich biological network constructed by mining PubMed abstracts
Source: BMC Bioinformatics. 2004 Oct 8;5:147. doi: 10.1186/1471-2105-5-147 (PMC528731; doi:10.1186/1471-2105-5-147)
Supplement: Additional File 2 — The original results of the above study (non-essential files are deleted to keep the file size under the limit set by BMC bioinformatics). [file 1471-2105-5-147-S2.bz2 › chilibotAdditionalFile2/dip40/65ID7729427E225/html/TAF12_TBP.html]

 


 **TAF12** and **TBP** 
  
Found 58 abstracts in PubMed, retrieved 40.  
 

 What does Google say? 
 PDF only 
| .edu only 

---

**Interactive relationship** (e.g. stimulation, inhibition, etc)

**Neutral relationship**- Substitution mutations of a glutamine rich region in one Sp1 activation domain, which eliminate interactions with a  **TBP**  associated factor  [ **TAF12** ] , also significantly decrease synergy with Tat.  Ref: 9400595 Virology, 1997
- The stimulation of Sp1 transcriptional activity by Rb is conferred, in part, through a direct interaction with the  **TBP**  associated factor  [ **TAF12** ]  TAF250.  Ref: 9926939 Oncogene, 1999
- Polymerase Pol III TATA box binding protein  [ **TBP** ]   **TBP**  associated factor  [ **TAF12** ]  Brf binds to a surface on  **TBP**  also required for activated Pol II transcription.  Ref: 9488486 Mol Cell Biol, 1998
- The human RNA polymerase II transcription factor B TFIID  [ **TBP** ]  consists of TATA binding protein  [ **TBP** ]   **TBP**  and the  **TBP**  associated factor  [ **TAF12** ]  TAF TAF II 170 and can rapidly redistribute over promoter DNA.  Ref: 11585931 Mol Cell Biol, 2001
- The human transcription factor B TFIID  [ **TBP** ]  is comprised of TATA binding protein  [ **TBP** ]   **TBP**  in complex with one  **TBP**  associated factor  [ **TAF12** ]  TAF of 170 kDa.  Ref: 9342322 Proc Natl Acad Sci U S A, 1997
- Furthermore, as Rep78 is known to bind at least one other transcription factor Sp 1 and likely others, Rep78 may function as a  **TBP**  associated factor  [ **TAF12** ]  in an altered TFIID  [ **TBP** ]  like complex.  Ref: 9614873 Virology, 1998
- The host factors that are known to regulate  **TBP**  activity include phosphorylation by host kinases and association with different  **TBP**  associated factor  [ **TAF12** ]  TAF subunits.  Ref: 11287600 J Virol, 2001
- Finally, our results suggest that  **TBP**  associated factor  [ **TAF12** ]  components of SAGA are differentially required for  **TBP**  binding to SAGA dependent promoters.  Ref: 12370284 Mol Cell Biol, 2002
- We demonstrate, utilizing a temperature conditional mutant allele of the gene encoding TAF25p, that this non histone like  **TBP**  associated factor  [ **TAF12** ] , which is shared between the TFIID  [ **TBP** ]  and SAGA complexes, is required for bulk mRNA gene transcription by RNA polymerase II in vivo.  Ref: 10383379 J Biol Chem, 1999
- The TATA binding protein  [ **TBP** ]   **TBP**  associated factor  [ **TAF12** ]  TAF II 250 is the largest component of the basal transcription factor IID TFIID  [ **TBP** ] .  Ref: 10648598 Mol Cell Biol, 2000
- The Epstein Barr virus EBV encoded transcriptional activator Zta recruits the general transcription factors IID TFIID  [ **TBP** ]  and IIA TFIIA to promoter DNA and induces a TATA box binding protein  [ **TBP** ]   **TBP**  associated factor  [ **TAF12** ]  dependent footprint downstream of the transcriptional initiation site.  Ref: 9343426 Mol Cell Biol, 1997
- Recently we have shown that T antigen is tightly associated with TFIID  [ **TBP** ]  and, in this position, performs a TATA binding protein  [ **TBP** ]   **TBP**  associated factor  [ **TAF12** ]  TAF like function.  Ref: 9632777 Mol Cell Biol, 1998
- In addition, NuA3 contains the  **TBP**  associated factor  [ **TAF12** ] , yTAF II 30, which is also a component of the TFIID  [ **TBP** ] , TFIIF, and SWI SNF complexes.  Ref: 10817755 Genes Dev, 2000
- The MADF domain directs sequence specific DNA binding to a site consisting of multiple trinucleotide repeats, while the BESS domain directs a variety of protein protein interactions, including interactions with itself, with Dorsal, and with a  **TBP**  associated factor  [ **TAF12** ] .  Ref: 12459265 Gene, 2002
- The TATA box binding activity of transcription factor IID TFIID  [ **TBP** ]  is autoinhibited by the N terminal domain of the Drosophila TATA box binding protein  [ **TBP** ]   **TBP**  associated factor  [ **TAF12** ]  230 yeast  **TBP**  associated factor  [ **TAF12** ]  145 subunit, which binds to the TATA box binding domain of  **TBP**  by mimicking the TATA box structure.  Ref: 10852950 Proc Natl Acad Sci U S A, 2000

**Non-interactive relationship** (e.g. studied together, co-existance, homology, etc.)

- One such repressed gene was identified as a TATA binding protein  [ **TBP** ]   **TBP**  Associated Factor  [ **TAF12** ]  TAF family member, TAF II 32, previously reported to be upregulated during apoptosis.  Ref: 10448067 Biochem Biophys Res Commun, 1999
- Transcriptional activation by c Jun depends on the TATA binding protein  [ **TBP** ]   **TBP**  associated factor  [ **TAF12** ]  TAF subunits of transcription factor IID TFIID  [ **TBP** ] .  Ref: 11316804 J Biol Chem, 2001
- Our studies indicate that Ii induced B cell maturation involves activation of transcription mediated by the NF kappaB p65 RelA homodimer and requires the B cell enriched coactivator  **TBP**  associated factor  [ **TAF12** ]  II 105.  Ref: 11371575 J Biol Chem, 2001
- It has been demonstrated that the inclusions in dentatorubral pallidoluysian atrophy DRPLA and Machado Joseph disease MJD were immunopositive for several transcription factors such as TATA binding protein  [ **TBP** ]   **TBP** ,  **TBP**  associated factor  [ **TAF12** ]  TAF II 130, Sp1, cAMP responsive element binding protein CREB and CREB binding protein, suggesting that neuronal degeneration in polyglutamine diseases may result from nuclear depletion of transcription factors containing the glutamine rich domain.  Ref: 11211058 Neuropathology, 2000
- The localization of the TATA binding protein  [ **TBP** ]   **TBP**  associated factor  [ **TAF12** ]  II70 TAFII70 in the germinal vesicle GV of newt oocytes was investigated.  Ref: 11768213 Genome, 2001
- Determination of the crystal structure of the human  **TBP**  associated factor  [ **TAF12** ]  hTAF II 28 hTAF II 18 heterodimer shows that these TAF II s form a novel histone like pair in the TFIID  [ **TBP** ]  complex.  Ref: 9695952 Cell, 1998
- TFIID  [ **TBP** ]  and SAGA contain common TATA binding protein  [ **TBP** ]   **TBP**  associated factor  [ **TAF12** ]  TAF II subunits and each complex contains a subunit with histone acetyltransferase activity.  Ref: 10864329 Nature, 2000
- Interestingly, a set of genes affected in the taf1 DeltaTAND mutant is similarly affected in the  **taf12**  HFD mutants but not in the nsl mutants of  **TBP** .  Ref: 12582246 Nucleic Acids Res, 2003
- In vitro, holo TFIID  [ **TBP** ]  and  **TBP**  associated factor  [ **TAF12** ]  250 TAF II 250 phosphorylated TFIIA on the beta subunit.  Ref: 11278496 J Biol Chem, 2001
- Mutations in the histone fold domain of the  **TAF12**  gene show synthetic lethality with the TAF1 gene lacking the TAF N terminal domain TAND by different mechanisms from those in the SPT15 gene encoding the TATA box binding protein  [ **TBP** ]   **TBP** .  Ref: 12582246 Nucleic Acids Res, 2003
- E7 is capable of recognizing a variety of proteins, including RB105, TATA box binding protein  [ **TBP** ]   **TBP** ,  **TBP**  associated factor  [ **TAF12** ]  TAF II 110, E2F, cyclins A and D, and c jun.  Ref: 10881991 J Hum Virol< MedlineTA>J Hum Virol, 2003
- The class II trans activator CIITA interacts with the  **TBP**  associated factor  [ **TAF12** ]  TAFII32.  Ref: 9171108 Nucleic Acids Res, 1997
- Electrophoretic mobility shift assays and DNase I footprinting revealed that acetyl CoA increased the affinity of the general transcription factor TFIID  [ **TBP** ]  for promoter DNA in a  **TBP**  associated factor  [ **TAF12** ]  TAF dependent manner.  Ref: 10688640 Mol Cell Biol, 2000
- We obtained a recessive insertion mutation in the gene encoding yeast  **TBP**  associated factor  [ **TAF12** ]  yTAFII61 68 that impairs Gcn4p independent and Gcn4p activated HIS3 transcription.  Ref: 9844640 Mol Cell, 1998
- Inhibition of host transcription by vesicular stomatitis virus involves a novel mechanism that is independent of phosphorylation of TATA binding protein  [ **TBP** ]   **TBP**  or association of  **TBP**  with  **TBP**  associated factor  [ **TAF12** ]  subunits.  Ref: 11287600 J Virol, 2001
- We observed efficient binding of a glutathione S transferase GST Gcn4p fusion protein to components of three different coactivator complexes in Saccharomyces cerevisiae cell extracts, including subunits of transcription factor IID TFIID  [ **TBP** ]  yeast TAFII20  [ **TAF12** ]  yTAFII20, yTAFII60, and yTAFII90, the holoenzyme mediator Srb2p, Srb4p, and Srb7p, and the Adap Gcn5p complex Ada2p and Ada3p.  Ref: 9488488 Mol Cell Biol, 1998
- A composite nuclear export signal in the  **TBP**  associated factor  [ **TAF12** ]  TAFII105.  Ref: 11567023 J Biol Chem, 2001
- Developmental regulation of a specific class of genes may therefore be achieved through changes in the availability of a  **TBP**  associated factor  [ **TAF12** ] .  Ref: 9642284 J Biol Chem, 1998
- Of these 400, interferon regulatory factor 4 IRF4, cyclin B2,  **TBP**  associated factor  [ **TAF12** ] , eukaryotic elongation factor and pim 2 were up regulated more than 3.5 fold.  Ref: 11790884 Dis Markers, 2001
- Transcription factor IIA derepresses TATA binding protein  [ **TBP** ]   **TBP**  associated factor  [ **TAF12** ]  inhibition of  **TBP**  DNA binding.  Ref: 9603936 J Biol Chem, 1998
- Coexpression of the human TATA binding protein  [ **TBP** ]   **TBP**  associated factor  [ **TAF12** ]  28 hTAFII28 with the altered specificity mutant  **TBP**  spm3 synergistically enhances transcriptional activation by the activation function 2 of the nuclear receptors NRs for estrogen and vitamin D3 from a reporter plasmid containing a TGTA element in mammalian cells.  Ref: 10373554 Mol Cell Biol, 1999
- The next gene in this map, located distal to SS18, was found to be the  **TBP**  associated factor  [ **TAF12** ]  TAFII 105 TAF2C2.  Ref: 11435705 Cytogenet Cell Genet, 2001
- These results demonstrate for the first time that a discrete fragment of a mammalian  **TBP**  associated factor  [ **TAF12** ]  which targets a specific activator can impair the stimulation of transcription.  Ref: 9311891 J Virol, 1997
- Here we describe the cloning and biochemical characterization of an abundant human  **TBP**  associated factor  [ **TAF12** ]  TAF 172 which is homologous to the yeast Mot1 protein and a member of the larger Snf2 Swi2 family of DNA targeted ATPases.  Ref: 9488487 Mol Cell Biol, 1998
- Requirement of tissue selective  **TBP**  associated factor  [ **TAF12** ]  TAFII105 in ovarian development.  Ref: 11557891 Science, 2001
- In this study, 14 3 3 proteins are shown to bind the TATA box binding protein  [ **TBP** ]   **TBP** , transcription factor IIB TFIIB, and the human  **TBP**  associated factor  [ **TAF12** ]  hTAF II 32 in vitro but not hTAF II 55.  Ref: 10449590 Plant Cell, 1999
